# Subsidies for Consumptives

**Published:** 1919-04-26

**Authors:** 


### SUBSIDIES FOR CONSUMPTIVES.

At a recent conference at the Sanitary Institute, Dr. Noel D. Bardswell, medical adviser to the London Insurance Committee, pointed out the fact, too often ignored, that consumptives, even after partial or complete arrest of the disease, cannot compete in the labour market with sound men. It is well known that the permanence of arrest of tubercular disease depends very largely on the life led by the consumptive after his discharge from a sanatorium, and that overwork, or worry, or underfeeding, or bad habits, speedily cause a recrudescence of the disease. But it is not sufficiently recognised that a consumptive is very easily overworked. His heart is under the influence of toxins; his temperature is readily raised; his digestion easily upset; and even healthy work of a comparatively light nature often leads quickly to a breakdown. Too often patients are advised to do farm work or gardening, simply because such work is work in the open air; but farm work is heavy work, and though it may be a heresy to say so, light indoor work is better for the patient than heavy work out-of-doors. Certainly a patient would have a better chance working as a clerk in a well-ventilated office and getting good wages for his work than doing a labourer's work on a farm and earning low wages. Unless in the case of patients with a private income, the question of wages must be considered; for wages mean food, and food is the sheet-anchor of the consumptive; and the question comes to be largely an economic question.

It comes, in fact, to be a question whether the patient can make a living without killing himself; and to this question the answer is that the great majority of "cured" consumptives cannot make a living without paying for the living with their lives. We have seen hundreds of consumptives go back to work and literally work themselves to death, and work themselves all the more quickly to death, if they have tried to make a good living by manual labour in the open air.

The "cured" consumptive, then, cannot maintain himself. The sooner this is recognised the better. Having recognised it, we must decide how to act in the face of such facts. Is it worth while arresting the disease at all? Would it not save the patient pain and the community expense if we made no effort to fight the disease?

No, even apart from humanity, from the lower level of economics, it will be found wiser to arrest the disease, but only if we recognise that in future the man has not full man-power and must receive economic assistance. The impairment of a man's working capacity must depend largely on his occupation and his educability. A navvy will not be a ninth part of a navvy after arrest of consumption; while a famous golfer after arrest of the disease continued his career as a "professional." Men with light and healthy occupations may not have their working and earning power much diminished; but in ninety-nine per cent. of cases there will be impairment of working and earning capacity, and if the man is to be kept for many years alive he will require some kind of subsidy to enable him to hold on to life.

The apportionment of subsidies will of course be an exceedingly difficult task, and probably only doctors of great sanatorium experience will be capable of judging the amount of work a consumptive can do without danger. But difficulties must not daunt us; for after-treatment with subsidy and supervision is the only solution of the problem of tuberculosis. After-treatment, subsidy, and supervision, moreover, will render it possible to control in some measure the domestic life of the patient, and to insure that he does not become a focus of infection, as is so often the case when discharged patients relapse, gravitate to the slums, and die. How far the principle of subsidy will eventally have to be carried it is difficult to say; but there can be no doubt that it will have to be extended to other patients than consumptives, for it is pretty certain, for instance, that a considerable proportion of "cured" shell-shock cases will also be liable to relapse and will be unfit to compete with men of sound nerves.

